# Community-informed perspectives of implementing interpersonal psychotherapy for couples to reduce situational intimate partner violence and improve common mental disorders in Mozambique

**DOI:** 10.1017/gmh.2024.92

**Published:** 2024-10-21

**Authors:** Jennifer J. Mootz, Palmira Fortunato dos Santos, Leyly Moridi, Katia dos Santos, Myrna Weissman, John L. Oliffe, Sandra Stith, Saida Khan, Paulino Feliciano, Antonio Suleman, Stephanie A. Rolin, Ali Giusto, Milton L. Wainberg

**Affiliations:** 1 Department of Psychiatry, Columbia University, New York, NY, USA; 2 Division of Translational Epidemiology and Mental Health Equity, New York State Psychiatric Institute, New York, NY, USA; 3 Department of Mental Health, Mozambique National Institute of Health, Maputo, Mozambique; 4 School of Global Affairs, Yale University, New Haven, CT, USA; 5 Department of Mental Health, Mozambique Ministry of Health, Maputo, Mozambique; 6 School of Nursing, University of British Columbia, Vancouver, BC, Canada; 7 Couple and Family Therapy Program, Kansas State University, Manhattan, KS, USA

**Keywords:** LMIC, CFIR, domestic violence, common mental disorders, interpersonal psychotherapy

## Abstract

**Background:**

High rates of intimate partner violence (IPV) and mental disorders are present in Mozambique where there is a significant treatment gap. We aimed to report Mozambican community stakeholder perspectives of implementing couple-based interpersonal psychotherapy (IPT-C) in preparation for a pilot trial in Nampula City.

**Methods:**

We conducted 11 focus group discussions (6–8 people per group) and seven in-depth interviews with key informants in mental health or gender-based violence (*n* = 85) using purposive sampling. We used grounded theory methods to conduct an inductive coding and then deductively applied the consolidated framework for implementation research (CFIR).

**Results:**

For the outer setting, local attitudes that stigmatize mental health conditions and norm IPV as well as an inefficient legal system were barriers. Stakeholders expressed high acceptability of IPT-C, although a lack of resources was a structural challenge for the inner setting. Adaptation of the approach to screen for and address potential mediators of IPV was important for adopting a multisectoral response to implementation and planning. Delivering IPT-C in the community and in collaboration with community stakeholders was preferable.

**Conclusion:**

Stakeholders recommended multilevel involvement and inclusion of community-based programming. Task shifting and use of technology can help address these resource demands.

## Impact statement

Sub-Saharan Africa suffers from the highest rates of intimate partner violence (IPV), a strong risk marker for common mental disorders, globally. High rates of IPV and mental disorders are present in Mozambique where there is a significant treatment gap. Efforts to reduce IPV often focus only on one type of IPV (intimate terrorism) and, even then, generally fail to include men unless court mandated. In high-income countries, state-mandated treatment for IPV perpetrators that involves gender-specific *men-only* treatment is generally ineffective but remains widespread due to concerns of victim advocates that couples-based treatment might provoke or exacerbate violence. Little information exists about the feasibility of a couples-based approach to reduce situational IPV and to improve mental health in low- and middle-income countries (LMICs). Moreover, scaling treatment for common mental disorders across Mozambique has not yet considered coordinated treatment of common mental disorders and the co-occurring problem of situational IPV among couples, a gap that could be addressed by adapting and implementing IPT for couples (IPT-C), a promising approach to reduce situational IPV (discord between intimate partners that can escalate to mild or moderate IPV) given one of its foci is to resolve disagreements. Safety components were integrated into IPT-C from *Couples Therapy for Domestic Violence: Finding Safe Solutions.* Through application of qualitative methods and use of the consolidated framework for implementation research to guide analysis, we elicited Mozambican community stakeholder perspectives of and recommendations for implementing IPT-C in preparation for a pilot trial in two hospitals in Nampula City. To the best of our knowledge, this is one of the first studies to evaluate community-informed perspectives and recommendations for implementing a couple-based therapy to resolve situational IPV and improve common mental disorders in an LMIC.

## Introduction

Globally, one of five people meets criteria for having a common mental disorder, such as depression, anxiety or post-traumatic stress disorder (PTSD), in the prior 12 months (Steel et al., 2014). Women are disproportionately affected and almost twice as likely to be diagnosed with a mood or anxiety disorder (Steel et al., 2014). Intimate partner violence (IPV) is a strong risk marker for common mental disorders among women (Trevillion et al., 2012; Howard et al., 2013). The association between IPV and severity of depressive symptoms is so strong that addressing IPV has effects on depressive symptoms comparable to those of short-term psychotherapy treatment for depression (Tsai et al., 2016). One in three women worldwide report lifetime exposure to IPV, and Sub-Saharan Africa suffers from the highest prevalence of IPV globally (44% of women) (Sardinha et al., 2022).

In 1995, Michael Johnson proposed that there are two types of IPV: situational IPV and intimate terrorism. Intimate terrorism is generally perpetrated by men and often includes severe, life-threatening violence that exhibits a pervasive pattern of domination and control of one’s partner using violence, characterized by fear (victim) and control (perpetrator). This contrasts with situational IPV that occurs when discord and conflict in couples escalates to mild or moderate physical violence (Johnson, 1995). Johnson (2006, p. 18) proposed that in situational IPV “the core problem is one of communication skill deficiencies for which an individual compensates with verbal aggression that then escalates into violence.”

### The present study

Given the mental health implications of IPV, the World Health Organization has recommended addressing IPV as a part of mental health services (World Health Organization, 2010). Yet, implementation of evidence-based treatments (EBTs) for IPV has been limited (Babcock et al., 2004). A recent meta-analysis, however, has shown some novel interventions, such as acceptance and commitment therapy, to have promising effect sizes when treating IPV perpetration (Babcock et al., 2024). Mozambique, classified as a low-income country, has a high prevalence of IPV (Andersson et al., 2007; Zacarias et al., 2012) and common mental disorders, (Vos et al., 2012; Audet et al., 2018) both of which are often untreated due to a mental health treatment gap resulting from a severe shortage of providers (Schwitters et al., 2015). Initiated in 2016, *Fica Bem* (“Stay Well”) is a collaborative implementation project with the Mozambique Ministry of Health and academic partners in the US, Brazil and South Africa to scale comprehensive care for mental health and substance use disorders by task shifting (i.e., using nonmental health professionals to deliver care) (authors blinded).

At the initiation of *Fica Bem*, the Mozambique team selected interpersonal counseling, a brief version of interpersonal psychotherapy (IPT) that is typically implemented in primary care settings (Weissman et al., 2014), to comprehensively treat common mental disorders. IPT has demonstrated effectiveness for treatment of common mental disorders in over 140 trials (Weissman et al., 2018), including in sub-Saharan Africa (Bolton et al., 2003; Bass et al., 2006) where IPT has high cultural relevance (Weissman & Mootz, 2024; Bass et al., 2006). IPT treatment centers on helping patients resolve an interpersonal problem (disagreements, grief, life changes or loneliness) and/or change their orientation to the problem (Weissman et al., 2018). The model integrates relational and attachment theory and research on social support and stress to assert that interpersonal disruptions explain psychiatric illness (Lipsitz and Markowitz, 2013; Weissman et al., 2018). This model is congruent with the concept of situational IPV as a chronic interpersonal disruption that increases women’s risk for common mental disorders (Howard et al., 2013; Biaggi et al., 2016; Tsai et al., 2016).

Scaling treatment for common mental disorders across Mozambique has not yet considered coordinated treatment of common mental disorders and the co-occurring problem of situational IPV among couples, a gap that could be addressed by adapting and implementing IPT for couples (IPT-C). The scale-up of IPC and other interventions through the Fica Bem study has initiated in Nampula Province. A national cadre of IPC trainers and supervisors has been built. Through the national IPC training program, we learned that participants often had disputes with intimate partners, but IPV was not systematically measured. Thus, in preparation for a pilot trial to test the acceptability and feasibility of implementing IPT-C to reduce common mental disorders in women by addressing situational IPV in couples, our team conducted pre-implementation qualitative research with the primary aim of obtaining community stakeholder input on barriers, facilitators and recommendations for implementing IPT-C.

Recognizing the limitations of individual IPT, IPT has been adapted for couples based on findings that 40% of female patients identified dispute with an intimate partner as the interpersonal problem area associated with onset of depression and that women with depression and marital discord exhibited poorer individual outcomes for depression than women who were in supportive relationships or single (Foley et al., 1989; Weissman and Klerman, 1993). IPT has been adapted for couples in the US to reduce postpartum depression (Carter et al., 2010). In China, couple-based IPT has effectively reduced postpartum depression symptoms in both mothers and fathers (Ngai and Gao, 2022). IPT has reduced depressive symptoms in women with histories of IPV in high-income countries with infrastructure to separate women from abusive partners (Cort et al., 2014). In many low-resource settings, however, numerous infrastructural barriers to separation exist and sociocultural norms discourage separation (Mootz et al., 2020).

Given safety concerns regarding working with couples who report any kind of IPV, special safety considerations and intervention components from *Couples Therapy for Domestic Violence: Finding Safe Solutions* (Stith et al., 2011) were integrated into IPT-C. Examples of these techniques include careful screening to insure the couple is appropriate for conjoint treatment, incorporating pre- and post-session check-ins with individual partners to assess safety, including psychoeducation about IPV as part of treatment, helping couples identify when anger and conflict escalate and helping them learn and practice strategies that facilitate de-escalation. Participants in IPT-C will be carefully screened to determine that they experience mild to moderate situational IPV.

### Couple-based interventions for IPV in low- and middle-income countries

To the best of our knowledge, only a few studies in low- and middle-income countries (LMICs) have adopted a couples-based approach, in essence, involving both partners in joint sessions to work on improving the couple relationship (Baucom et al., 2014), to reducing IPV and little information exists about the feasibility of a couples-based approach in these settings. The Safe at Home pilot trial in the Democratic Republic of Congo tested effectiveness of weekly single-sex discussion groups and monthly family (couples and children) discussion groups about gender, power, violence and parenting in community settings to improve family functioning and reduce IPV and harsh discipline against children. While family functioning did not significantly change, they found that Safe at Home effectively reduced victimization and perpetration of IPV (Falb et al., 2023). A study in Rwanda provided an intervention called Indashyikirawa, consisting of group (usually gender-mixed) training sessions for couples affiliated with local Village Loan and Savings Association groups to reduce gender-based violence (GBV) and improve couple relationships (Dunkle et al., 2020). The authors of Indashyikirawa found that, when compared to a control group, the intervention effectively reduced IPV victimization and perpetration (Dunkle et al., 2020). A study in Zambia showed reductions in IPV through HIV prevention counseling of couples (Jones et al., 2014). Other research in Zambia tested the Common Elements Treatment Approach to reduce IPV among couples and men’s alcohol use (Murray et al., 2020). While couple members were treated individually, the study found that the intervention was more successful in improving these problems than treatment as usual (Murray et al., 2020). Two additional IPV studies among couples have taken place in India. The first study implemented one session with couples and two sessions with men individually and found that women reported experiencing less sexual IPV at the 18-month follow-up assessment (Raj et al., 2016). Another cognitive behavioral therapy intervention in India was conducted with couples where male partners were using alcohol. This intervention showed significant reductions in IPV following initial sessions with men to reduce alcohol and four subsequent sessions with couples to improve communication (Hartmann et al., 2021).

Beyond these studies, efforts to reduce any kind of IPV often fail to include men unless court-mandated, an uncommon procedure in low-income settings, even though couple-based IPT has been shown to be effective in resolving disputes among couples (Foley et al., 1989; Weissman and Klerman, 1993). In high-income countries, state-mandated treatment for IPV perpetrators that involves gender-specific *men-only* treatment is generally ineffective but remains widespread due to concerns of victim advocates that couples-based treatment might provoke or exacerbate violence (Armenti and Babcock, 2016). Conversely, couple-based mental health EBTs often exclude couples who experience IPV (Jiwatram-Negrón and El-Bassel, 2014). This gap could be addressed by carefully screening couples to determine that the IPV they are experiencing is situational and by engaging both partners in mental health treatment in a couple modality (Mootz et al., 2024; Armenti and Babcock, 2016; Karakurt et al., 2016).

### Implementation science framework

To tackle public health crises, such as widespread IPV and high prevalence of common mental disorders, interactions and underlying sociopolitical vulnerabilities that give rise to co-occurring disorders must be addressed (Singer et al., 2017). Our team conducted a systematic review of interventions for family violence and co-occurring conditions in LMICs and found that none of the 19 studies across 13 LMICs had explicitly applied an implementation science lens (Mootz et al., 2023). Others have also stated that implementation science studies targeting IPV are lacking (Kim, 2021). Prefaced on the criticality of the pre-implementation phase for optimal implementation and tailoring of the delivery of IPT-C to fit the Mozambican context, we used the consolidated framework for implementation research (CFIR) to capture contextual variables and determine strategies to affect multiple domains and processes (Damschroder et al., 2022). The CFIR compiles implementation constructs using information from multiple sources to examine barriers to and facilitators of care in five domains: innovation (what is being implemented); outer setting (sociopolitical context, in this case, Mozambique); inner setting (setting where innovation is implemented, e.g., two hospitals in Nampula city); individuals (characteristics and roles) and implementation process (strategies for implementation) (Mootz et al., 2024; Damschroder et al., 2022).

## Methods

### Procedures

We focused our qualitative data collection in Nampula City, given that the pilot trial will occur in two public hospitals there. The primary outcomes of the pilot trial will be acceptability and feasibility, although preliminary outcomes of common mental disorder symptoms, perpetration and victimization of IPV, and quality of the couple relationship will be evaluated. Nampula City is in Nampula Province, a northern province comprised of 23 districts. It is the most populous province of Mozambique. The majority of the population in Nampula Province lives in poverty, and the literacy rate, access to safe water and access to health facilities is lower than the national average (UNICEF, 2022). Men in Nampula are twice as likely than women to be literate. There are 236 operational health facilities across the province: one regional, six at the district level, two rural hospitals, 204 primary clinics, 24 secondary clinics and two categorized as other (World Health Organization, 2022).

We adopted a policy- and community-partnered, participatory approach through assembly of a Community Advisory Board (CAB) that consisted of hospital community liaison representatives, Ministry of Health personnel and GBV service experts. We also created a policy- and community-partnered technical team with national leaders and local, Nampula-based expert trainers and supervisors in interpersonal counseling and its scale-up across Nampula. We obtained ethical approval for the project in Mozambique through the Comité Nacional de Bioética em Saúde and at an academic institution in the US.

With input from members in the CAB and community-partnered technical team, participants were recruited through word-of-mouth and from hospital waiting room areas. Groups were structured according to community partners’ recommendations (e.g., having gender-segregated groups of men and women given the sensitive topic areas) of various stakeholders who could provide different perspectives about implementation according to the CFIR domains, such as policymakers, providers and potential recipients of the intervention. KS, a female psychologist and the project coordinator, led 11, 45–60-min focus group discussions (FGDs; ranging from 6 to 8 people per group) with separate groups of women (*n* = 15; two FGDs) and men (*n* = 6; one FGD), experts in GBV (*n* = 7; one FGD), providers (*n* = 20; three FGDs), CAB members (*n* = 8; one FGD), district officials (*n* = 7; one FGD), community leaders (*n* = 8; one FGD) and elders (*n* = 6; one FGD) (Mootz et al., 2024). Mental health and GBV providers were recruited from the Nampula Psychiatric Hospital and Nampula Central Hospital. First, the research coordinator contacted the participants a week before the interview. FGDs were done at the Nampula Psychiatric Hospital and individual interviews were done at the Nampula Central Hospital. All FGDs and interviews were completed after the end of providers’ workday. The Research Coordinator and research assistants contacted the Régulo of Nampula (a formal position responsible for community leaders in Nampula), presented the study, and he helped identify community leaders and elderly from diverse neighborhoods in Nampula to participate in FGDs. For inclusion, participants needed to be age 18 or over and/or had a professional role representative of various included stakeholders. Anyone demonstrating psychosis, inebriation or cognitive impairment was excluded.

Three individual interviews were done at the start of the study to maintain social distancing in adherence to COVID-19 regulations. An additional five key informant interviews were conducted with IPV or mental health policymakers. FGDs and interviews continued until data saturation was reached, determined through team discussion in weekly meetings.

The FGDs and key informant interviews began by having the research assistants obtain signed consent forms and gather demographic information from the participants. During these audio-recorded discussions and interviews, one or both research assistants were present to record notes. The project coordinator facilitated these conversations in Portuguese using a semi-structured interview guide organized around various domains of the CFIR that was developed by the PI, reviewed by the project team and iteratively adapted throughout data collection. Following a description of IPT-C, central topics of discussion related to perceptions of IPT-C, questions about engaging men, and obstacles and supports for implementation. Throughout the interviews and FGDs, the interview guide was adjusted. This involved simplifying existing questions and eliminating redundant questions. Three different versions of the interview and FGD guides were developed for key informants, community members and officials and providers.

### Data analysis

Transcripts were transcribed verbatim by two research assistants. A team of four, consisting of one U.S. researcher and three local research team members, collaborated in developing the codebook. Initially, open codes were assigned to sections of narrative that focused on specific topics, forming meaning units. These initial open codes were discussed in biweekly meetings. The next step involved the U.S. researcher developing a preliminary codebook in Portuguese from transcripts that had been translated into English (the U.S. researcher’s first language), which was then discussed in meetings by the four team members. This discussion aimed to clarify any confusion and add new codes as necessary and drive consensus.

Subsequently, the local team members uploaded the codebook into an online qualitative coding software, Dedoose. The coding process involved the two local research team members and the project coordinator working and coding together, ensuring full agreement through discussion before assigning codes to excerpts. Weekly meetings with the fourth U.S. researcher helped address any questions, and the codebook was continuously updated.

Following the coding phase, the team, in collaboration with another local researcher, organized the codes into themes known as axial codes (Corbin and Strauss, 2008) over 2 days of a data analysis workshop. Assignment into axial codes was decided through discussion and consensus of the group. These codes, along with their frequency of application according to Dedoose, were then compiled into a table. The team engaged in discussions to conceptualize how codes could be combined and identified which codes emerged consistently across FGDs and interviews. Any exceptions, such as those mentioned only once or twice, were also considered.

These themes were then further organized through team discussions into an overarching thematic framework through a process known as selective coding. The team presented this overall thematic framework separately to the community-partnered technical team and the CAB during in-person meetings. The purpose of these meetings was to seek input on the relevance and face validity of themes, given their experiences working with the community. The use of multiple analysts, multiple groups of stakeholders, use of FGDs and interviews, and obtainment of input from the community-partnered technical team and the CAB both strengthen the credibility of the study through triangulation (Corbin and Strauss, 2008).

## Results

### Participant characteristics

Eighty-five participants took part in the study, with more than half (55%) identifying as women and most as being of Black African descent (95.3%). A small number identified as White (2.4%), mixed race (1.2%) or having other ethnic backgrounds (1.2%). Ages of participants spanned from 18 to 75 years, with an average age of 38.9 years. Most participants (91%) had children and most (89%) lived with a partner or partner and children. In terms of educational attainment, half of the respondents had completed secondary education, 29.8% held a university degree, 11.9% had only primary education, 3.6% possessed a post-graduate degree and 4.8% reported no formal education. All participants were of Mozambican nationality and the majority (72.6%) were from Nampula Province. There was a diverse range of activities among the participants for employment: 50% were engaged in formal employment, 13.1% were involved in domestic work, 11.9% were self-employed, 9.5% pursued various roles such as community leadership or activism, 6% were unemployed, 4% worked as volunteers and 4% were studying.

### Implementation barriers, facilitators and recommendations

Results are organized below by the themes that emerged relevant to the CFIR domains (see Table 1). Within each section, we use italics to denote the specific constructs within each domain described by participants. While all constructs mentioned are documented in Table 1, the results below summarize the central themes.

**Table 1.** Implementation considerations for IPT-C according to the CFIR

CFIR domain	Construct	Topics	Participant
Innovation	Evidence base	Emerging research area; IPT-C presents opportunity for evidence-building	Community leaders, key informants
	Relative advantage	IPT-C preferred over family- and community-based interventions; few to no programs in health sector exist for combined approach to reducing situational IPV and mental health	Women, providers, CAB, key informants
	Complexity	Implementation considered feasible; however, limited specialists available	Key informants, GBV experts, women
	Cost	Recommend no patient payments; leverage community structures to reduce campaign costs	Women
Outer setting	Local attitudes	Sociocultural considerations: *Potential barriers* - women’s financial dependence on male partners, gender norms that discourage reporting IPV, stigma related to mental health problems and mental health care settings. *Potential facilitator* - women offer support to one another	Women, providers (three FGDs), elders, CAB, key informants, men, community leaders, GBV experts, district officials
	Local conditions	Stakeholder engagement including government and Ministry of Health, civil society, religious/spiritual leaders, community are important and should be considered.	GBV experts, CAB, key informants, providers, men, district officials, providers
	Partnerships and connections	Community stakeholders and structures in place and legal authorities are important and should be leveraged.	Providers (two FGDs), key informants, CAB, community leaders
	Policies and laws	Laws and policy to protect people from IPV exist but may not be reinforced. Reporting to legal authorities usually reserved for severe cases and there’s fear of reporting.	Providers (three FGDs), key informants, district officials, community leaders, CAB, men, women
Inner setting	Communications	Hospital-based violence services have a flow of communication in place with police.	Key informants, CAB, woman, providers
	Compatibility	Existing violence and outpatient mental health services in the clinic setting can be leveraged to deliver the intervention.	Providers, CAB, key informants
	Incentive systems	Some providers expressed difficulty of not being valued.	Providers
	Available resources	Barriers in terms of physical infrastructure (private office space), work infrastructure (specialized mental health providers) and information technology infrastructure (electronic health records).	Community leaders, providers, key informants
	Access to knowledge and information	Specialized psychologists are present in these settings, which can facilitate implementation.	Key informants, district officials, elderly, providers (three FGDs), community leaders, CAB, GBV experts
Individuals	Innovation deliverers	Capability: specialized psychologists; request for additional, ongoing training Need: ongoing, specialized training needed Motivation: providers expressed high acceptability of delivering the intervention	CAB, providers (three FGDs), district officials, key informants, women, community leaders
	Innovation recipients	Need: high prevalence of many forms of violence. Observation that violence induces physical, emotional injuries. There’s a cyclical, generational impact of witnessing violence. Related needs are substance abuse, having a positive HIV status.	Women, providers
Implementation process	Teaming	Adopt a multisectoral approach with religious leaders and organizations, civil society, local and national government, legal authorities, mental health specialists, community leadership and grassroots participation; integrate mental health specialists into GBV services	GBV experts, key informants, community leaders, providers (three FGDs), CAB, men, women, district officials
	Planning	Multisectoral engagement beneficial; include those with connection to the community; develop an evidence base	CAB, providers, district officials, key informants
	Engaging innovation recipients	Increase community awareness to improve engagement; address stigma related to men’s help-seeking; deliver the intervention locally	GBV experts, CAB, men, women, key informants, providers, community leaders, elders
	Reflecting and evaluating	Benefit of consistent patient follow up for monitoring and evaluation	Providers (two FGDs), key informants
	Adapting	Recommendation to adapt to address mediating factors and other problems related to IPV; include community and neighbors to try to support couples	Providers (two FGDs), community leaders, CAB, key informants, women

*Note*: CFIR, consolidated framework for implementation research; IPT-C, interpersonal psychotherapy for couples; IPV, intimate partner violence.

#### Innovation

Participants across several stakeholder groups (e.g., women, providers, CAB, key informants) described a pathway of how IPV broadly is typically responded to in the community. This starts with a family-based response, often led by male relatives, as the most common recourse for any couple conflict, including when IPV is present. If the conflict cannot be resolved, a neighborhood secretary is consulted for advice. Finally, in extreme instances, legal recourse is sought. A member of the women’s FGD explained the standard family procedure for responding to marital conflict in the following excerpt:

In case my husband continues, or I continue to do something, we go out to the family, I don’t go to my family, but to my husband’s uncle’s house and I tell him everything. He comes and advises his nephew. In case he doesn’t listen to his uncle, I go to my family. That’s how I solve it. – FGD, women.

Participants asserted that attempts to resolve IPV within the family are often insufficient, with one provider explaining these practices may reinforce the idea that a woman is obligated to accept violence as a normative byproduct of marriage. A CAB member explained the following:

There are different psychoeducational models, and one of the models is the more traditional model where families try to reach an understanding between them based on, we would call it, the ideas or opinions of more influential people, people with more experience in solving these cases, which ends up harming those directly involved in the violence. – FGD, Community Advisory Board.

Participants also highlighted the absence of current interventions for IPV within the healthcare system. They noted the lack of instruments to facilitate identification of IPV and emphasized the need for dedicated IPV programs. Several participants expressed discontent with current efforts to reduce IPV and, as a result, were enthusiastic about introducing innovative interventions, viewing this as having *relative advantage* to the current response in communities and the healthcare system. A key informant perceived IPV not to be a priority for the public health system and referenced a pilot intervention designed to address GBV, which was experiencing delays in implementation.

The lack of effective resources for reducing IPV, in addition to the norming of GBV, were recognized as factors contributing to common mental disorders among women. GBV experts, key informants and women acknowledged the *complexity* of addressing IPV. As a member of the women’s FGD shared, “It is very complicated. We really need to have other ideas to face this situation.” Additional supports including the police was suggested as necessary. Community leaders and key informants expressed the need for more evidence to support the effectiveness of current interventions and did not raise concerns with the *evidence base* of IPT-C for reducing situational IPV and common mental disorders. One community leader, for example, advocated for developing a standardized process for addressing IPV, given this is a new research area in Mozambique, reflecting a desire for empirically driven approaches to IPV prevention and support. Notably, however, this topic was not raised by providers.

Women mentioned *cost-*related aspects. They were in agreement that the intervention cost should be minimal to none. A woman from the community implied that male recipients may be unwilling to spend money on the innovation, wherein maintaining low or no out-of-pocket payments should be a priority. Perhaps this topic was not mentioned by other stakeholders due to mental health services being public and available for no cost.

#### Outer setting

All FGDs and key informant interviews discussed *local attitudes* at length, usually as impediments to help-seeking for any kind of IPV and mental health services. Stigma toward help-seeking for IPV was grounded in gender norms and relations for women to be submissive to male partners, an expectation that hinders them from reporting IPV. Men, on the other hand, were perceived as often having dominant roles in the family, and any act of reporting them was seen as a defiance in transgressing those cultural norms. A member of the GBV expert FGD explained, “What makes me a woman is the fact that I am there to help my husband.” Participants explained that women who report abuse are often stigmatized and discredited by being labeled as gossipers, leading to isolation and ostracism. Participants indicated there is a prevalent belief that women who complain about or report their husbands do not truly love them. Women who do report IPV sometimes experience pressure from their community to drop the case. This GBV expert shared:

When one goes out and goes to report it, all the others will say, “You don’t love your husband. You are not a real woman. What kind of woman are you to denounce your own husband?” … The neighborhood just hears that she reported it. She is so pressured in the neighborhood that sometimes the woman goes back to the police and says, “I want to drop the case. We already have come to an understanding.” – FGD, GBV experts.

In addition to sociocultural gendered hierarchies and pressures, there are also practical reasons why women may be less likely to seek help for IPV. Women’s financial dependence on men was often cited as a significant reason for their silences about IPV and/or avoidance of taking any actions toward leaving abusive situations.

If my husband beats me, I won’t report it. I can’t put my husband in jail because he’s the one who feeds me. If I complain about my husband, what are you going to feed me? He’s my husband. I shouldn’t say anything. He feeds me. – FGD, GBV experts.

Regarding mental health, cultural messages advise women to be “resilient” and bear problems silently, causing these problems to go unaddressed. Mental health symptoms, like depression resulting from IPV, are often ignored or misunderstood. Survivors of IPV are sometimes viewed as going through a phase and not genuinely suffering from significant psychological distress.

If it is a woman who is a victim of physical violence and she has anxiety or depression, the community treats her as if you were going through a difficult phase in your marital life and they try to give you the support, the “that’s how it is” support. – Key informant.

Mental health problems remain heavily stigmatized in Mozambique, which may adversely affect potential recipients’ willingness to participate in the intervention. One provider stated that seeking treatment from a psychologist is commonly perceived as an option reserved “for the crazy.”

Despite the ubiquitous conversation about stigma, participants also described a willingness of neighbors and friends to be sources of support. Neighbors can offer insight into problems and symptom manifestation experienced by individuals for broader perspective. Emotional support can be reciprocal whereby women can comfort one another. A woman explained her experience in the following narrative:

She tends to give affection, for example, there’s a neighbor of mine, when I’m really bad, she comes to comfort me. I can say that we’ve gotten used to it, she and I. We support each other a lot. Sometimes she’s the one who has nerves. We talk, and we calm down. – FGD, Women.

Multiple participants suggested the idea that a broader community, including friends and neighbors, could empower women facing these challenges. A provider suggested, “We could also involve the patient’s neighbors and friends to help empower the patient.” Yet, there remained concerns about lack of confidentiality and stigma from the surrounding community for those facing IPV and mental health problems.

Most FGDs and key informants also had several recommendations for stakeholder engagement for critical *partnerships and connections.* Several people mentioned importance of including religious leaders, for example. The excerpts suggested a need for a collective response that involves different sectors of society and multiple stakeholders, described in the quotation below, to address complex issues like IPV and mental health.

The involvement of all stakeholders, if we are talking about violence, violence is not just a case that is resolved by the health sector or by the Interior Ministry so that everyone is involved, including the Provincial Secretariat, the Government, the State must be involved because the problem of violence is a common problem. It is a problem of society. And Civil Society and the Human Rights League must also enter this planning, which is to be an activity to be implemented jointly, cohesively, and has roots. – Key informant.

Participants involved in service delivery (i.e., providers, GBV experts and key informants) described it as necessary to have governmental involvement at all levels in policymaking, monitoring and intervention implementation. Top-down engagement could ensure policies have the necessary impact and scale. A provider explained, “In the design of policies, the government must be aware if this program is involving a lot of people…” One participant expressed concern that failure to achieve buy-in from the Ministry of Health would severely jeopardize the implementation process.

If the Ministry of Health doesn’t take this package so seriously it won’t work, it is necessary that the big decision makers were involved and aware of this program. It would be very important. – FGD, providers.

There was also discussion about ensuring a flow of information from the topmost level to the grassroots implementers, involvement of cross-sectoral governmental bodies, and having multisectoral teams in the communities to address issues holistically.

All FGDs and key informants apart from elders observed that IPV survivors who seek help are faced with several institutional and political roadblocks in *policies and law.* Two community health committee members raised impunity and the failure of the judicial and police systems in ensuring that perpetrators of GBV are “punished in accordance with the law.” Participants emphasized the importance of public institutions in reducing IPV, with one provider acknowledging the Mozambican government’s efforts to install a secretariat responsible for women’s and gender-related issues.

#### Inner setting

When discussing *structural characteristics*, the theme of infrastructure and *available human resource* constraints emerged as the most prevalent and recurring one. Community leaders, providers and key informants consistently expressed was the importance of privacy through the provision of separate and discreet spaces for therapy, consultations and treatments. The desire is for spaces where couples can discuss their issues without the fear of being overheard, judged or shamed. The participants also emphasized the benefits of privacy in facilitating open and honest communication. A key informant noted that health units had been built many years prior and may not reflect the current need for distinct office space. Moreover, participants thought that health units were not structurally equipped to handle the patient inflows for their existing services.

In fact, the most difficult thing here in Nampula is how spaces are laid out, because there are a lot of inflows in the health units, this is a fundamental aspect that could make the implementation of the intervention more difficult. If there was a larger space and the inflows were well structured, perhaps this issue could be well resolved to be able to support all the patients who are in the health unit, as well as human resources as well. – FGD, district officials.

Although most participants agreed that providing a private, designated space for delivering the intervention would be critical, opinion was divided as to whether the service should be provided in a hospital setting. Some expressed that the existing mental health workforce and public health structures should provide solid foundations for the intervention. Several other participants felt that hospital settings provide minimal comfort and privacy, and that the stigma surrounding receiving treatment in hospitals could deter recipients. Participants recommended using a specialized mental health office to deliver services discreetly. Suggestions also included placing mental health specialists in all health units and engaging community leaders in awareness and support efforts. However, a key informant stated that health units are “minimally prepared” for delivering IPT-C, and that hospitals would be a more appropriate location for the intervention.

I think there has to be a place with more privacy, a specific place for this type of situation, for people to feel comfortable talking to the provider, in a pleasant and favorable environment, so that the person feels good because… in hospitals there is not that secrecy component, so to speak. It is a case of the person having more privacy and not being exposed by their problem. – Provider.

Providers, members of the CAB, and key informants highlighted that existing violence and mental health services are in place, making IPT-C *compatible*, and that there are existing channels of *communication* regarding these issues. They expressed positive opinions of health units’ workflow practices, including the use of “service flowchart[s]” (district official) and “triage [systems]” (key informant) facilitated by psychologists to ensure that patients are referred to the correct services following an initial consultation. A member of the GBV expert FGD recommended opening an additional office dedicated to IPV, with personnel to “serve users well, secretly, confidentially.” Yet, human resource constraints in the public health system were identified as an obstacle to intervention delivery. Participants expressed concern that bureaucracy and limited provider capacity could lead to long patient wait times.

The inadequate access to information technology also emerged as an inner setting barrier. One participant explained that providers often have trouble accessing patients’ medical history and are therefore less well positioned to make informed decisions about appropriate treatment. Introducing streamlined electronic records would improve the continuity of service provision, particularly when a patient is attending an appointment with a new provider.

He spoke of a very important aspect, the electronic records…The [medical] history will help the provider in making a decision about that situation, having a computer recording system that the patient arrives and already has his [patient ID] for example…[having] all the information will also make things easier for the provider who is handling that case for the first time. – FGD, providers.

As potential barriers, provider confidence and lack of *access to knowledge and information* were raised. Offering training to both midlevel providers and psychologists could compensate for the limited human resource across health units. Moreover, participants noted the role that multisectoral collaboration could play in delivering training to providers. While incentives and reward mechanisms were not discussed explicitly, several participants indicated morale as a potential barrier, with one provider stating, “It is very annoying when you work so much but your work is not valued.”

#### Individuals

##### Innovation deliverers

Across interviews and FGDs, there was high acceptability toward the intervention. A district employee FGD member stated, *“*I think this IPT-C approach for couples is a good initiative. Our communities are prepared to receive everything*.”* Participants demonstrated positive attitudes toward combating situational IPV and CMDs, in addition to providing recipients with impartial and confidential services. Demonstrating *motivation* to engage, overall providers welcomed the intervention and expressed strong interest in participating. They often described the intervention as a tool that can help address the issue of situational IPV.

I don’t know, is there any scale? From zero to ten, I’m on ten, from zero to a thousand, so I’m so confident…I think that any psychologist or provider would be very happy to have one more tool that will help. – FGD, providers.

There was consensus among most FGDs and interviews that specialized providers were *capable* of delivering IPT-C as long as the *need* of ongoing, specialized training was met. A district employee FGD member said, “I think that every provider is capable of dealing with this intervention, but it depends on their ability to know the cause or matter*.”* They highlighted the significance of provider professionalism and noted that recipients of the intervention may feel vulnerable if their provider was a member of their local community.

##### Innovation recipients

Women and providers explicitly confirmed a *need* for an intervention that addresses situational IPV and common mental disorders through articulation of various physical and emotional injuries – depression, sadness, trauma and isolation – that result from exposure to numerous forms of IPV prevalent in the community. They additionally highlighted the generational impact of witnessing violence as a child and the risk of becoming a perpetrator of violence as an adult. A member of the women’s FGD observed, “The child, in turn, will be watching the behavior of the parents, then they will follow the same method as the parents*.”* The most cited types of violence were physical, psychological and economic, oftentimes forms of violence were related as the following excerpt illustrates.

An example is when my neighbor receives a salary, he does not buy food at home, he only buys alcohol, then he beats his wife and to feed the children she asks for food from the neighbors. – FGD, providers.

#### Implementing

Almost all FGDs and key informant interviews highlighted the importance of pursuing a multisectoral *teaming* approach to implementation. Participants identified the government, civil society and health sectors as key stakeholders for the intervention. Leveraging and strengthening existing collaborations between the Office for the Integrated Assistance of Victims of Violence (Integrated Service Centers are based in hospitals and provide medical care for GBV victims and access to legal and police services), the Ministry of the Interior, and the health sector, was viewed as crucial to the sustainability of the intervention. Participants also indicated buy-in from local stakeholders was critical to the implementation process. Suggestions frequently included training community leaders, including religious and spiritual leaders, and utilizing their proximity to recipients to improve trust in the intervention.

Cross-sectoral collaboration was also an important part of *planning.* Most FGDs and interviews recommended involving key stakeholders, including families with IPV and mental health providers, in the initial planning stages.

I don’t know who the people are, but at this planning stage it was important to include some families who are already going through a situation of the same nature, then other tools could be added to first prepare. – FGD, providers.

Participants indicated it would be beneficial to deploy representatives from sectors with greater connection to locals than mental health professionals, such as “regulators and police officers,” to lead community sensitization efforts (provider). Evidence gathering was also mentioned as an important factor in the planning process to ensure that the objectives and goals of the intervention are realistic given baseline data.

I think it would be necessary first to make a baseline, to try to find out what are the objectives, the possible results that are intended to be achieved in this IPT-C strategy and then having the objectives and results to be achieved prioritizing the places of intervention. – FGD, GBV experts.

A lack of providers could affect provider consistency, something that was stressed as being important for reducing unnecessary anxiety and preventing delays in understanding the full scope of recipients’ needs.

If I’m having a session with a certain therapist, it’s good for me to continue with the same therapist until the end, because there is already discomfort on the part of the couple. In the first session it’s with a certain therapist, then the second one goes to another, and you’ll have to summarize everything you said in the first session. I don’t think that makes for good consistency. – FGD, providers.

Providers and key informants highlighted that maintaining consistent follow-up appointments with couples would ensure the wellbeing of recipients and help *evaluate* whether the intervention was successful in the longer term.

If I, as a therapist, am following a patient, I have to monitor it, for example: I am following a certain patient who I have scheduled a certain date for follow-up, if that patient does not appear to me, I cannot rest assured, I would have to find out where is, as it is, even if it’s over the phone. This would also help the patient to think “Hey, someone cares about me,” and to look for [seek] the health unit more. I think this could help. – FGD, providers.

Providers, community leaders, CAB members, key informants, and women raised the importance of *adapting* the intervention content to the local context and addressing mediating factors to IPV, including HIV status and substance use among men. Many participants suggested using the therapy sessions to screen for alcohol use, which they viewed as common among men, although the men’s FGD did not raise this as an issue. If detected, psychologists may then use the sessions simultaneously to raise awareness among the couple about the relationship between alcohol use and situational IPV.

In a couple therapy session on alcohol consumption, where it is detected, we can talk to the partner in all sessions. In the first session we must try to understand what exactly motivates the partner to act aggressively and after identifying the problem, we can recommend that they stop drinking alcohol. We know that it is very difficult for the partner to reduce the excessive consumption of alcohol. – FGD, district officials.

One participant acknowledged that IPT-C sessions could be beneficial for gaining a deeper understanding into alcohol overuse and for motivating recipients to reduce their alcohol intake (district official).

Several participants acknowledged that responding to the mental health needs of men would be challenging due to the stigma surrounding mental health issues in Mozambique.

For me, it is essential to work with men first, because if a man manages to commit an act of violence, it is because he is the first one who is not well. The man is the first one who is not well, because someone who is well psychologically, because someone who is well with himself will not at any time harm another person, he will not reach the point of hitting his wife. – FGD, GBV experts.

Increasing community awareness of IPV was broadly viewed by most stakeholder groups as crucial for *engaging intervention recipients.* For example, one participant recommended developing audiovisual campaigns to disseminate information accessibly.

There needs to be community awareness, but the other part is that this awareness can be used through the media, for example, making audiovisual recordings where this new service can be publicized and the message can reach people who often don’t bring with you time to listen to a certain person in a setting like this, but wherever they are, whether at work or at home, people can take advantage of those messages that are transmitted there or decimated from there on. – FGD, GBV experts.

Participants also recommended delivering the intervention locally to increase accessibility and service utilization. A community leader referenced a previous initiative to hold HIV mobile testing clinics in schools which had successfully increased testing among men in the community, and could serve as a model example for delivery.

Substantial discussion focused on improving male engagement in the intervention, with participants indicating that deconstructing *machista* attitudes toward men’s behavior and mental health should be a priority. (See authors blinded for a more in-depth discussion of masculinity and male engagement.) One participant noted that men may be reluctant to participate due to fear of legal repercussions.

Another difficult aspect lies in convincing the violator, because he is very afraid, we have experience because we hold lectures in places where there is always a violator and when talking about something that he is perpetrating or has already done, he feels afraid, he thinks that this to be searched or after here they will put me in jail. – FGD, GBV experts.

Respondents typically suggested that women engage their male partners. A woman shared, “We can go try to pull our husbands*.”* Another woman elaborated, “For me, I can only find ways to have a dialogue with men, to try to explain better so that they understand so that they can address this therapy.” A member of the men’s FGD concurred and succinctly stated, “This man will come to therapy only with the woman’s help.” A provider affirmed that the conversation must start at home.

## Discussion

To the best of our knowledge, this is one of the first studies to evaluate community-informed perspectives and recommendations for implementing a couple-based therapy to resolve situational IPV and improve common mental disorders in an LMIC. It includes perspectives of community-based women and men, GBV experts, district officials, providers, community leaders and elders to effectively implement IPT-C in an urban setting in Mozambique. The findings underscore enthusiasm for the intervention and recommendations for implementation that span 25 constructs of the five CFIR domains.

Participants described a perceived lack of effectiveness in long-standing pathways for addressing conflict among couples (usually a family intervention, with escalation to health and legal services only in cases with high severity). While participants did not label this type of couple conflict as situational IPV, it is likely that most IPV would fit within this conception and the severe cases that present at the hospital more likely to fall under intimate terrorism. The limitations of traditional crises driven IPV responses have been described in other sub-Saharan contexts. For instance, a qualitative study consisting of interviews with IPV survivors and perpetrators in the Democratic Republic of Congo found that this type of intervention typically construed women as responsible and advised them to find ways to be deferent to their male partners (Kohli et al., 2015). Another qualitative study with 25 female survivors of IPV in Zimbabwe reported a similar emphasis on resolving couple conflict within family first and that this approach was a barrier to help-seeking as disclosing IPV to non-family-members was stigmatized (Chadambuka and Warria, 2022). Given these considerations, stakeholders in this study were therefore eager to have a tool to address IPV within the context of the couple. However, given the severity of cases that present to the hospital, it may be less likely that a couple-based therapy would be appropriate due to safety concerns.

The sociocultural environment in the outer setting of the intervention presents challenges for those experiencing these conditions. Stigmatization of help-seeking for any kind of IPV, discussed by participants, is well-documented in the literature including gender norms that impede women from reporting IPV (Overstreet and Quinn, 2013). Participants in our study noted social repercussions for reporting IPV and difficulties due to their financial dependency on their male partners. This finding aligns with others who have similarly documented economic inequities among women and men as a critical barrier to addressing IPV (Chadambuka and Warria, 2022). Given these challenges, many IPV survivors prefer to preserve the relationship, rather than separate and pursue legal avenues, in order to protect their family’s ability to meet basic needs (Kohli et al., 2015; Chadambuka and Warria, 2022). Using data from almost 5,000 women in Mozambique, researchers identified that religious norms and presence of early marriage in a region were strong sociocultural predictors of women’s accepting attitudes of IPV (Cau, 2020). As the recommendation to include religious and spiritual leaders was common in this study, consideration for how best to include them in ways that do not deter women’s engagement or reinforce stigma in the implementation strategy would be fruitful next steps. One possibility could be to train religious and spiritual leaders to deliver the intervention or, depending on time availability, identify someone else who could facilitate such an intervention for couples. Other strategies could be to focus on strengthening referral pathways between religious institutions and mental health care.

In addition, since men experience social and self-stigmas for mental health help-seeking, research participants recommended destigmatizing campaigns to mobilize community support. This was suggested to lessen isolation and encourage couples experiencing IPV to seek support outside of their familial network. Throughout our research, participants offered examples of social support garnered from neighbors and friends that they described as beneficial. A query using the CFIR- Expert Recommendations for Implementing Change (CFIR-ERIC) (Waltz et al., 2019) tool confirmed that participatory inclusion of patients, families and community boards would be fruitful. Overall, decreasing stigma through participatory involvement and awareness campaigns can increase possibilities for support garnered from community.

As providers are embedded within specific sociocultural contexts, it is unsurprising that participants in this study emphasized a need for providers to be welcoming, nonjudgmental and impartial. Providers are not immune to prevailing attitudes about IPV and mental health providers can sometimes provide care that is influenced by these biases. Other studies from LMICs have confirmed that acceptance of violence and negative attitudes toward IPV survivors among providers, is a barrier to help-seeking and delivery of care (Colombini et al., 2017; Schwab-Reese and Renner, 2018). Suggestions to combat this have been to offer providers ongoing, specialized training (Colombini et al., 2017), a recommendation also made by participants in this study who stressed that training and dissemination of information were crucial for program success. Given that ongoing provider training can be a costly, resource-intensive activity, finding ways to reduce costs, such as using interactive phone- or web-based modalities, would help support feasibility and sustainability, especially in LMICs.

Participants emphasized the importance of considering sociocultural context and frequently suggested to include community members and leaders in a community-based delivery approach. A scoping review of 31 IPV screening, management and treatment programs in low-resource areas, with most studies from Africa, highlighted program implementation barriers being low cultural sensitivity and relevance (Schwab-Reese and Renner, 2018). The authors cautioned the dangers of not adapting to context by describing a project where all participants left the study because of a lack of cultural sensitivity. This is a poignant reminder that community-based participatory work in the areas of IPV and mental health is not just recommended, it is essential. A review of 29 articles of community-based mental health interventions in post-conflict, LMIC settings found that facilitators of community-based approaches were adopting approaches that were task-shifted (delivered by nonspecialized providers), transdiagnostic (treating more than one mental illness concurrently) and customized to context (Al-Tamimi and Leavey, 2022). Thus, training lay or peer providers to implement IPT-C for transdiagnostic problems (e.g., depression, anxiety and PTSD) using community-based implementation strategies (e.g., in people’s homes) could optimize facilitation.

While many preferred a community-based implementation option, others noted that the inner hospital setting was compatible, although less scalable, with existing mental health and IPV services and described channels of communication that already exist between these services. A systematic review of 11 studies in LMICs of IPV programming in the health sector identified many factors as facilitators to integration, such as presence of well-defined guidelines, intersectional coordination and established referral pathways. The presence of facilitators operating together allowed for the most well integrated responses (Colombini et al., 2017). Another review in sub-Saharan Africa of eight IPV interventions confirmed the importance of providing comprehensive services beyond screening and having established referral pathways. The authors noted a barrier in the referral process where specialized services were not accessible and linkage to further care was poor. Some in this study suggested having psychologists in all health units and engaging community leaders in awareness and support efforts. Barriers in the inner setting included infrastructure and human resource constraints, recurrent themes in mental health intervention literature in LMICs (Qureshi et al., 2021), which could improve consistency of care and ability to adequately evaluate programming. Use of technology to track patient outcomes, referral pathways and linkage to care could help provide continuity in low-resource settings. Participants’ emphasis on privacy and the adequacy of current health facilities resonates with the pervasive structural challenges found in mental health service delivery in LMICs.

Addressing the complex nature of IPV and common mental disorders necessitates adopting a multisectoral response. Many participants named policy and governmental services as essential partners for sustained implementation of programming. To identify implementation gaps for IPV services, Jethá et al. (2021a) analyzed 11 national policy or guideline documents in Mozambique and conducted individual interviews with IPV experts. They identified primary gaps as follow through with holding perpetrators accountable as well as providing specialized training for providers. Other apparent gaps were the need to establish a data management, monitoring and evaluation system (Jethá et al., 2021b). Participants in our study also identified a need for monitoring and evaluation, a process they suggested could be strengthened through consistent follow-up by providers, a challenge due to the shortage in the specialized workforce.

Finally, the need to tailor interventions to the local context, addressing concurrent issues like substance use and HIV, is a recurring recommendation in the field. The participants’ suggestions for a comprehensive response that incorporates these factors are in line with an integrated approach to IPV interventions. A review of integrated IPV and HIV studies from sub-Saharan Africa found that seven out of the nine interventions had been implemented in community-based settings and several effectively improved community-level HIV knowledge and social empowerment for women (Anderson et al., 2013). However, our systematic review of interventions targeting family violence and mental health or substance use problems concluded that interventions and their effectiveness varied widely, and more effectiveness research is needed (Mootz et al., 2023). Adaptations for couples, however, should consider that male alcohol use is highly related to IPV, as participants remarked. Implications for programming are questions related to what degree substance use, and what levels, would interfere with effectiveness of an IPV and mental health intervention, and the service delivery flow of how to address these comorbid problems. For example, delivery systems would need to consider whether men should participate in an individual substance use intervention first before conjoint therapy could be started, a strategy that could present barriers to overall engagement depending on men’s perceptions about levels of substance use and readiness to change.

## Limitations

Several limitations should be acknowledged in interpreting the results of our study. Our recruitment approach, though inclusive of multiple stakeholders, relied on word-of-mouth and recruitment from hospital waiting areas and community settings. This strategy may introduce selection bias, especially in hospital settings, as those present in the hospitals might possess different characteristics, experiences or opinions from the broader community or those not accessing hospital care. Additionally, there was no inclusion criteria that participants be currently partnered, which may have affected participants’ ability to discuss help-seeking for mental health and IPV. However, given that most participants lived with a partner or partner and children, it is likely that participants had experience with couple relationships. While the interview guide was structured, one primary facilitator who was also the project coordinator led the FGDs and interviews, which could have introduced interviewer bias and affected the dependability. Our analyses did not differentiate speaker by gender, an analysis that could have provided important insights. The participatory approach of engaging a Community Advisory Board and a community-partnered technical team, while strengthening the community-research partnership, may also introduce a form of response bias. Members who are closely involved in project implementation might have perceptions influenced by their involvement or may be hesitant to provide critical feedback. Although Nampula City was chosen for its relevance to the pilot trial, it remains a specific urban area within the broader context of the Nampula Province. The city’s characteristics and the selected study locations within public hospitals may not capture the diverse experiences and nuances found in other regions, particularly in more rural or different healthcare settings within the province. Although IPT-C is meant to address mild to moderate situational IPV, participants tended to talk about IPV broadly and did not differentiate between types of IPV.

## Conclusion

IPV is a pervasive, global issue with significant public health implications. Yet, implementation studies regarding IPV services are scant. This analysis examined the perspectives and recommendations of various stakeholders in Mozambique toward implementing couple based IPT to reduce IPV and improve mental health using the CFIR at the pre-implementation stage. The primary barriers were stigmatization and social norms regarding help-seeking for IPV and mental health in the sociocultural context and resource gaps in infrastructure at the inner setting level, the latter a barrier that could be addressed (at least in part) with investment in digitized technologies. While many perceived that the intervention was an improvement upon traditional family-based responses and that hospital services aligned with the delivery of the intervention, the need for strong community involvement and community-based delivery was underscored as key to feasibility and acceptability. Adapting the intervention to address related problems of substance use and HIV also seems key to the local context.

## Data Availability

Data are available from the corresponding author upon reasonable request.
