# Supplementary material for: Community-informed perspectives of implementing interpersonal psychotherapy for couples to reduce situational intimate partner violence and improve common mental disorders in Mozambique
Source: Glob Ment Health (Camb). 2024 Oct 21;11:e84. doi: 10.1017/gmh.2024.92 (PMC11504926; doi:10.1017/gmh.2024.92)
Supplement: Peer review history

# Editor report: Recommendation: Community-informed perspectives of implementing interpersonal psychotherapy for couples to reduce situational intimate partner violence and improve common mental disorders in Mozambique — R0/PR2

**DOI:** 10.1017/gmh.2024.92.pr2

**Affiliations:** Director, National Institute of Mental Health, Czech Republic

No accompanying comment.

# Editor report: Decision: Community-informed perspectives of implementing interpersonal psychotherapy for couples to reduce situational intimate partner violence and improve common mental disorders in Mozambique — R0/PR3

**DOI:** 10.1017/gmh.2024.92.pr3

**Affiliations:** London School of Hygiene & Tropical Medicine, United Kingdom of Great Britain and Northern Ireland

No accompanying comment.

# Editor report: Recommendation: Community-informed perspectives of implementing interpersonal psychotherapy for couples to reduce situational intimate partner violence and improve common mental disorders in Mozambique — R1/PR5

**DOI:** 10.1017/gmh.2024.92.pr5

**Affiliations:** Director, National Institute of Mental Health, Czech Republic

No accompanying comment.

# Editor report: Decision: Community-informed perspectives of implementing interpersonal psychotherapy for couples to reduce situational intimate partner violence and improve common mental disorders in Mozambique — R1/PR6

**DOI:** 10.1017/gmh.2024.92.pr6

**Affiliations:** London School of Hygiene & Tropical Medicine, United Kingdom of Great Britain and Northern Ireland

No accompanying comment.

# Editor report: Recommendation: Community-informed perspectives of implementing interpersonal psychotherapy for couples to reduce situational intimate partner violence and improve common mental disorders in Mozambique — R2/PR8

**DOI:** 10.1017/gmh.2024.92.pr8

**Affiliations:** Director, National Institute of Mental Health, Czech Republic

Your paper will be accepted pending the incorporation of minor changes suggested by the reviewer.

# Editor report: Decision: Community-informed perspectives of implementing interpersonal psychotherapy for couples to reduce situational intimate partner violence and improve common mental disorders in Mozambique — R2/PR9

**DOI:** 10.1017/gmh.2024.92.pr9

**Affiliations:** London School of Hygiene & Tropical Medicine, United Kingdom of Great Britain and Northern Ireland

No accompanying comment.
